# Psychological adjustment of Spanish adolescents and their parents during COVID-19 lockdown: A mixed method approach

**DOI:** 10.1371/journal.pone.0255149

**Published:** 2021-07-29

**Authors:** Silvia Postigo-Zegarra, Martín Julián, Konstanze Schoeps, Inmaculada Montoya-Castilla

**Affiliations:** 1 Department of Psychology, Faculty of Health Sciences, Universidad Europea de Valencia, Valencia, Spain; 2 Department of Personality, Assessment and Psychological Interventions, Faculty of Psychology, Universitat de València, Valencia, Spain; IRCCS E. Medea, ITALY

## Abstract

Previous literature on the psychological impact of COVID-19 has shown a direct relationship between family conflicts and psychological distress among parents and their children during the domestic lockdown and social isolation; but there are also opportunities to enhance family bonding, encourage collective problem-solving and improve personal relationships. This study aimed to explore psychological adjustment processes of Spanish adolescents and their parents during the first month of lockdown by analyzing their narratives, perceived outcomes, protection and risk factors. A total of 142 people agreed to participate in this study. Of all participants, 61 were adolescents (*M* = 13.57; *SD* = 1.74; 57% women) and 81 were parents (*M* = 46.09; *SD* = 4.72; 91% mothers). All were Spanish residents and completed an online survey during the domestic lockdown in March 2020. From a qualitative design, methodology followed a mixed approach to analyze data. The results showed three different types of adaptation to lockdown and social isolation in both adolescents and their parents: 1) positive adjustment, 2) moderate adjustment, and 3) maladjustment. Most participants reported a good adjustment and only a 20% of parents and a 16% of adolescents stated that they had not been able to achieve a positive psychological adjustment. There are few significant quantitative differences between adolescents and their parents. The qualitative analysis of data showed that adolescents reported less psychological distress than their parents. The two most important protective factors were social support and keeping busy during lockdown. The most significant risk factors were loss of mobility and social isolation. The conclusions stressed that regarding psychological maladjustment, parents experienced feelings of uncertainty whereas adolescents experienced a kind of mourning process. These findings can be used to design and implement effective intervention measures for mental health and psychological well-being in such a difficult situation as domestic lockdown.

## Introduction

The rapid and unexpected outbreak of COVID-19 has led to a global pandemic [1] causing a disruption of the economic and health system around the world. In Spain, a state of emergency was declared on 14 March 2020 followed by a three-months lockdown [2]. During the first two weeks, people were only allowed to leave their home to buy essential groceries and/or going to their workplace. In the second two-week period, restrictions were tightened and non-essential work activities were suspended [3].

This unusual situation has resulted in the increase of various psychological problems such as depressive or post-traumatic stress symptoms, insomnia, irritability, anxiety, and feelings of loneliness [3–8]. However, simple coping behaviors such as keeping a healthy diet, following a daily routine, giving up on watching the news or updates on COVID-19 frequently, enjoying personal hobbies and staying outdoors or looking outside the window have been found to be the best predictors of low levels of anxious and depressive symptoms in Spanish adults [9].

Regarding adolescents and their parents recent research has indicated that they may have been particularly affected by the lockdown restrictions [10–15]. These state measures have increased the likelihood of interpersonal conflicts in many households. Both adolescents and their parents were forced to develop coping strategies to deal with an unusual daily routine of combining work, school and leisure activities and conducting all within one tight space [15, 16]. Additional struggles to families were caused by the possibility of having been infected by COVID-19, economic difficulties and living together under the same roof in a limited space [15]. Research on the impact of COVID-19 on parents has identified childcare as one of their major concerns during the pandemic. In addition, the poor economic and health situation of household members, the social isolation of their children and the schooling quality during the lockdown have been identified as some of the risk factors contributing to a highly demanding environment for parents [17]. Furthermore, family members who struggle with physical and/or psychological problems prior to the pandemic faced additional stressors that may aggravate the situation of the COVID-19 lockdown.

Recent scientific literature has reported several risk factors for adolescent mental health such as the length of the domestic lockdown period, boredom, fear of infection, lack (or excess) of information, loss of social interaction with peers and teachers, the utilization of negative coping strategies and lack of privacy at home [7, 10, 18–20]. According to a study conducted in Spain and Italy, two of the countries most affected by COVID-19 in March 2020, parents reported several psychological problems among their children. The most frequent symptoms observed among adolescents were concentration difficulties, boredom, irritability, restlessness, nervousness, feelings of loneliness, and COVID-19 related concerns [11, 21].

However, according to the perspective of the adolescents, many of them have developed the necessary skills to adapt to this unusual situation and learn to deal with the insecurity, manifesting even less anxiety than their parents [16]. Protection factors such as social support have proved to prevent anxiety and depressive symptoms in adolescents [12]. From a qualitative perspective, Italian adolescents reported several stressful experiences regarding the limitation of autonomy, the impact of a new life routine, the loss of social interaction with school peers and negative emotions. Furthermore, being part of an extraordinary experience, discovering oneself, rediscovering family life and sharing life at a distance have been the most positive experiences during the domestic lockdown [22].

With regard to effective family functioning, characteristics such as parents’ neurotic personality, which is considered as a set of concerns, emotional instability and feelings of inadequacy have led to an increase in parental stress and to children’s problematic behavior [23]. Similar, children’s behavioral patterns of hyperactivity, inattention and maladaptive emotional symptoms led to an increase in parental stress and higher levels of neuroticism among their parents during the lockdown. A dyadic research has stressed that a negative family climate and poor emotional regulation strategies among adolescents were critical in predicting parents’ emotional maladjustment while poor emotional regulation strategies among parents increased adolescents’ emotional distress [24]. Finally, spending more time with their children appeared to have a positive influence on some families considering the adverse impact of the restrictions [15, 17].

Previous scientific literature has shown a direct relationship between family conflicts, parental psychological distress and a wide range of psychological problems in adolescence during domestic lockdown due to COVID-19 [17, 23, 24]. Furthermore, some studies have reported a growth in opportunities to improve family bonding, encourage collective problem solving and improve personal relationships [15, 22]. However, to our knowledge, the psychological adjustment of adolescents and their families during lockdown have not been thoroughly explored. This might be an important limitation to understand how these negative and positive outcomes have risen and how they can be useful in preventing and treating further psychological problems. In addition, it could led to the promotion of family health.

Consequently, this study aims to explore the psychological adjustment of Spanish adolescents and their parents during the first month of lockdown due to COVID-19 by analyzing their narratives on the adaption process, the perceived outcomes and perceived protection and risk factors. At the end we asked families about their living experiences during the lockdown. We focused on the discourse in order to understand the perspectives of the participants and the importance of perceived protection and risk factors that made the adaption to the extraordinary situation easier or harder to bear.

## Materials and methods

### Participants

A total of 142 people agreed to participate in this study. Of all participants, 61 were adolescents (*M* = 13.57; *SD* = 1.74; 57% women) and 81 were parents (*M* = 46.09; *SD* = 4.72; 91% mothers). All were Spanish residents and were forced to stay at home during the national lockdown in March 2020. The number of families that included parent-child dyads was at least 37 since not all parents of participating adolescents and not all children of participating parents responded to the online survey. Most of the participants were living in Valencia (*n* = 122; 85.92%) and the rest in other Spanish regions (*n* = 22). All the participating families reported a medium socioeconomic level. Regarding their educational level, adolescents were attending 6th grade of primary school (*n* = 7; 11.48%), obligatory secondary school (1st grade: *n* = 11; 18.03%; 2nd grade: *n* = 9; 14.75%; 3rd grade: *n* = 20; 32.79%: 4th grade: *n* = 11; 18.03%) or 1st grade of post-compulsory education (*n* = 3; 4.92%). Most parents reported higher education (*n* = 47; 58.02%) and the rest received a secondary (*n* = 26; 32.10%) or primary education (*n* = 8; 9.88%). Only 9 parents (11.11%) reported that they have been infected with the COVID-19 at that time of the study (although 6 of them were not yet confirmed diagnoses), 17 parents reported some kind of physical illnesses such as asthma, allergies, hypertension or chronic pain (20.99%), 17 parents reported current or pre-existing psychological problems, usually anxiety or depression (20.99%) and 6 parents were undergoing psychological treatment at the time the study was conducted.

### Procedures

The research was approved by the Ethics Committee of the University of Valencia, which included the study protocol and the form of informed consent to participate in the study. Parents were recruited by E-Mail using the snowball sampling technique, and they were requested to recruit their own children for voluntary participation. The recruitment E-Mail included two links. One link was for the parents’ assessment protocol and the other link was for the children’s assessment protocol. Both were completed and registered through an online secured platform and preceded by the informed consent. Both parents and children were required to give their agreement to participate. The participants received information on the study including the main objective of the research and the anonymous and confidential treatment of their data, guaranteeing compliance with the human rights and the European General Data Protection Regulation. The survey was developed using the online platform Limesurvey and the access was available from March 26 to April 20 2020.

### Instruments

Sociodemographic data were collected by an *ad hoc* questionnaire including questions about age, gender, socioeconomic level and studies. Parents were required to provide information on prior or present psychological and physical illnesses including COVID-19.

An online qualitative survey with open-ended questions, answered and registered online, was used to collect data on the adaptation process to the domestic lockdown and social isolation. Online qualitative surveys offer several benefits to both researchers and participants such as the potential for rich and focused data, affordable and easy access to the large geographically dispersed populations, encouraging the revelation of sensitive topics, and noninvasive and flexible participation [25]. All participants (parents and adolescents) answered the same online survey on their own on electronic devices. The online survey was without a time limit. The research questions were: 1. *Tell us how are you dealing with the “Stay at home requirements”*?, 2. *What is helping you to deal better with the domestic lockdown* and 3. *What is the worst thing about the domestic lockdown*? The three open-end questions comprised the three main research questions: 1. Personal discourse or narrative of adaptation process; 2. Perceived protection factors, capturing things, behaviors or attitudes that make participants easier to adapt to the domestic lockdown and social isolation; and 3. Risk factors, capturing the most important constraints to successfully adapting to this situation.

### Data analysis

This research follows a qualitative and transversal methodology for exploratory purposes, in an empirical design with multiple case studies, employing a mixed method of data analysis, that is, integrating quantitative and qualitative analyses of the collected data [26]. To maximize the transparency on the method and data analysis, the report of this research follows the consensual guidelines from the Consolidated criteria for reporting qualitative research (COREQ) and the standards for reporting qualitative research (SRQR) [27–29].

For the analysis of the qualitative data, a content analysis was performed with a thematic coding strategy for the three open-ended questions using Excel and Atlas.Ti software [30, 31]. In addition, for the first open-ended question, which dealt with the adaptation process, a discourse analysis was carried out. To the responses given by the participants the syntactic articulation of "*doing well / doing just okay / not doing well*, *because*…" was adopted. This analysis permitted not only to identify regular patterns in the data indicating the outcome of the perceived adaptation process (the basic categories identified as a good, moderate, or bad adjustment), but also the perceived reasons for this outcome. The analyzed data were not returned to the participants for commenting or correction, neither was the online survey repeated.

Qualitative data were analyzed in a systematic and iterative manner. The analyses included several steps to reach data saturation and to maximize the internal validity or coherence of the results [30, 31]. First, two expert researchers independently conducted an initial content analysis of all three open-ended questions using thematic coding. They identified a flexible number of descriptive categories, which directly emerged from the text data. At this point, the analysis of the first research question (adaptation process) became a discourse analysis, whereas the other two questions were analyzed in the next step of content analysis.

For the discourse analysis of the first research question on the adaptation process, after coding each answer as positive, moderate or negative adjustment, the saturation of the data was discussed, and the core themes were identified. Interrater agreement was calculated based on the Cohen’s Delta model (Table 1), indicating a high concordance (Δ = .90 indicates that 90% of the answers are concordant and not by chance) [32]. Before conducting further analysis, the two coders reached full consensus on the core themes of the adaptation process (positive adjustment, resignation and maladjustment). In the second step, one of the coders analyzed the syntactic structure of the perceived outcomes, which have been described by the participants, and classified their responses into smaller categories. In the third step, the other researcher reviewed the resulting structure and categories. Finally, both researchers discussed the results in several meetings to achieve a full consensus on each assigned response and how the narratives of the adaptation process were articulated.

**Table 1:** Core themes for the study variables.

Core themes	Parents				dolescents				Z test	
	Cohen’s delta index		Frequency		Cohen’s delta index		Frequency			
	Δ^a^	*SE*^b^	*n*^c^	%^d^	Δ^a^	*SE*^b^	*n*^c^	*%*^d^	*Z*	*p*
**Perceived outcomes**										
**Doing well**	0.95	0.03	34	55.74	0.95	0.03	55	67.90	-1.48	.14
**Doing just okay**	0.94	0.04	15	24.59	0.91	0.05	13	16.05	1.27	.20
**Not doing well**	0.95	0.03	12	19.67	0.94	0.04	13	16.05	0.56	.58
**Protection factors**										
**Social support**	0.79	0.07	40	32.52	0.88	0.06	34	37.78	-0.79	
**Favorable circumstances**	0.95	0.03	9	7.32	0.88	0.06	3	3.33	1.24	.21
**Keeping busy**	0.82	0.06	40	32.52^e^	0.75	0.08	42	46.67^e^	-2.09	.03
**Time planning**	0.93	0.04	11	8.94	0.94	0.04	3	3.33	1.63	.10
**Perception of opportunities**	0.91	0.04	6	4.88	0.88	0.06	5	5.56	-0.22	.82
**Personal resources**	0.89	0.05	10	8.13^e^	0.94	0.04	1	1.11^e^	2.28	.02
**Civic responsibility**	0.91	0.05	5	4.07	0.94	0.04	0	0	1.93	.06
**Nothing**	0.95	0.03	2	1.63	0.94	0.04	2	2.22	-0.31	.74
**Total**			123	100			90	100		
**Risk factors**										
**Loss of social contact**	0.95	0.03	45	39.47	0.79	0.08	40	53.33	-0.07	.93
**Loss of mobility**	0.82	0.06	41	35.96	0.75	0.08	24	32.00	1.89	.06
**of freedom**	0.91	0.05	14	12.28^e^	0.94	0.04	1	1.33^e^	3.00	.00
**Negative emotions**	0.93	0.04	8	7.02	0.86	0.06	7	9.33	0.01	.99
**Problems of cohabitation**	0.91	0.04	5	4.39	0.82	0.07	1	1.33	1.49	.13
**Nothing**	0.95	0.03	1	0.88	0.94	0.04	2	5.67	-0.69	.48
**Total**			114	100			75	100		

^a^ Cohen’s delta of inter-rater agreement.

^b^ Standard error.

^*c*^ Number of references.

^d^ Percentage of references.

^e^ Significant differences between proportions (*p* < .05).

For the content analysis of the other two research questions, the second step was the same as above. The two coders independently classified the responses first into descriptive categories (directly emerged from the text data), and then into broader or basic categories, aiming to capture the core themes that describe perceived protective and risk factors. Interrater agreement was calculated using the Cohen’s Delta model, obtaining high concordance rates (Table 1). In the third step, the coders discussed the differences in the coding process using the *check- coding* procedure until they reached a satisfactory agreement rate.

In addition, we were interested in potential quantitative differences between adolescents and parents in their discourse on adaptation process, as well as perceived protective and risk factors. Thus, a two-proportion Z test was used to compare the proportion of broad or basic categories (themes) between the two groups in all three research questions. Specifically, we compared the number or proportion of textual references, which have been assigned to each of the categories, and whether there were differences between adolescents and parents to determine whether the discourse differed quantitatively. The null hypothesis for the z-test is that the proportions are the same at a significance level of 95%, the critical value being 1.96.

Finally, in order to discuss and give coherence to the reported results in relation to the reviewed literature, an interpretational analysis was carried out by two other members of the research team (not the coders). Thus, they compared the broad and basic categories, which have emerged from the collected data, with the empirical and theoretical results from previous research. In this study, analytical processes of coder triangulation, as well as theoretical triangulation were used to enhance the credibility and reliability of the qualitative data analysis, [26, 30]. The external and pragmatic validity of this research will be addressed later in the discussion.

## Results

Participants’ narratives of how they have adapted to the *“Stay at home requirements”* have been organized into three basic categories: doing well, doing just okay, not doing well. These categories are the same in adults and adolescents, allowing proportion comparisons between them (Table 1). However, the adjustment-maladjustment narrative is articulated in both populations from different core themes. As shown in Table 1, the majority of participants reported "doing well" not leaving home (*n* = 89; 62.38%), both parents (n = 55; 67.90%) and adolescents (*n* = 34; 55.74%). However, a significant number of participants reported "not doing well" (*n* = 25; 17.61%) or "doing just ok" (*n* = 28; 19.72%); not observing significant differences between parents and adolescents. The participants’ narratives regarding the aspects that are helping or hindering them to adapt to the domestic lockdown and social isolation are similar between adolescents and adults, and can be described through the same basic categories, making possible to carry out comparisons of proportions between both groups (Table 1) that are explained below.

### Parent’s discourse according to perceived outcomes

#### Positive adjustment: “Doing well”

**Fig 1:**
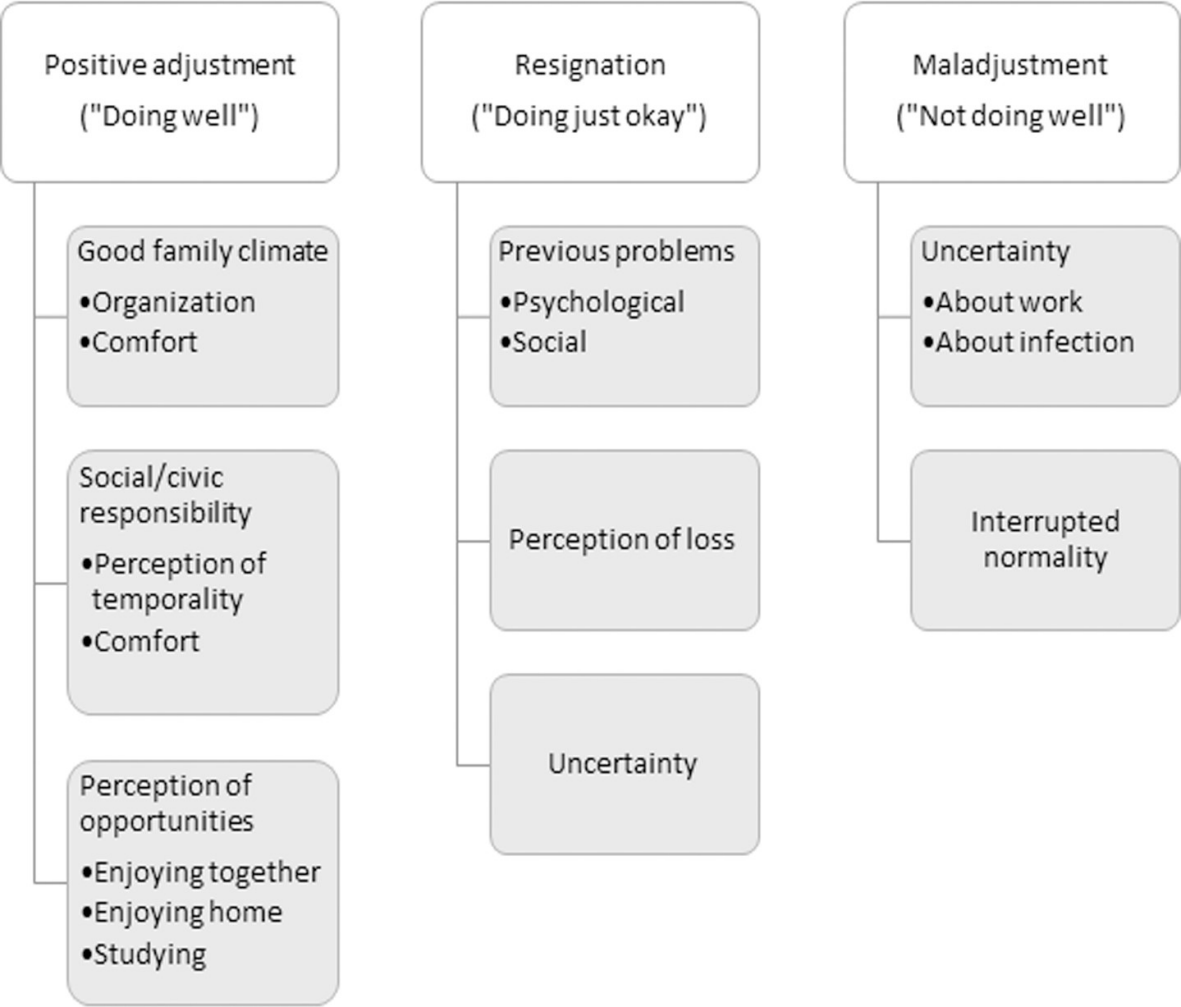
Core themes of parent’s discourse according to their perceived adaptation to confinement.

Among those who say that they are "doing well" or "better than they thought" not leaving the house, the discourse is articulated from different elements that suggest a positive adjustment (Fig 1). First, the majority of these parents reported time and efforts dedicated to create a **good family climate**, fostered by the organization at home, in terms of establishing new routines and organizing times and spaces: *"Well*, *we have established some routines at home and spending time together is very nice"*, *"Trying to organize our time”*, *“Good*, *we have organized a schedule that includes exercise routines and education"*, *"As we are all working online*, *we are all respecting each other’s space more";* and, in few cases, favored by the existence of certain amenities: *"Well*, *we have arranged the rooms quite well*, *lucky to have a townhouse with several floors"*.

Second, the awareness of **civic responsibility** and perception of temporariness seem to make easier to cope with the lockdown situation by providing a meaning for the effort and the restraints: "*I’m doing well*. *I am sure this is the best thing I can do for my own and for others*. *It’s something temporary"*, *" We are bearing it in a positive way with the perspective that it’s a hard but passing period and filling the domestic lockdown with awareness and responsibility (*…*) we will all be rewarded"*; *"I don’t mind not being able to leave the house because we have some comforts*, *and the situation requires it"*.

Finally, some parents also identify the domestic lockdown as an **opportunity** to make or enjoy things they usually don’t have time to do, as enjoying together: *"Now we are more united and do more things together"*, *"I enjoy my children more and spend more time with them*, *something I didn’t do due to my job"*, *"Being able to do things together that during the daily stress of work schedules and tasks we can’t do*"; enjoying the house: *"I enjoy doing chores around the house"*, *"I’m doing pretty well*. *I like being at home*. *I can’t usually be at home*, *so now I’m enjoying it"*; or even studying: “*I kill time by taking some courses I have pending”*.

Surprisingly, more than a half of the parents, who have reported pre-pandemic physical, psychological or social problems, have shown a positive adjustment. However, these parents also refer emotional ups and downs ("*sometimes I get overwhelmed"*, *"considering the bad situation*, *I am calm"*) that seem to be primarily related to the social situation rather than the family or personal situation: *"personally*, *I am doing well; collectively*, *I am not doing well"*, *"my spirit is low when I get in touch with the suffering of the people who are infected with the virus*, *who are close to infected people or those who have died*, *the health care professionals*… *causes me a lot of pain*, *impotence and helplessness"*. Other reasons mentioned for these ups and downs or occasional concerns are job-related *("at first it was bad because I didn’t have work and had to put my workers in a temporary dismissal"*), problems with cohabitation ("*at first I’m quite positive*, *but I’m not able to make my daughter follow routines and our relationship is tense which makes me feel sad"*), missing "*feeling the breeze in my face"*, and worrying about not being able to help loved ones, especially the elderly who don’t live in the same house.

#### Resignation or moderate adjustment: “Doing just okay”

Among those who are doing just okay, "*okay*", "*not so good*" or "*not so bad"*, the discourse is articulated from the feeling of "*riding a roller coaster*", that is, that *"sometimes I’m doing well*, *and sometimes I’m not"*. Thus, the ups and downs mentioned above are harder in this discourse and, in general, the mood was resignation. Parents who feel this way usually report some kind of **pre-existing problems** that have been enhanced by the domestic lockdown, including emotional problems such as anxiety: *“I’m doing passably*, *I am taking more anxiolytics*, *there are good days and bad days”;* or depression: *"I am used to it (not going out)*, *I am a housewife*, *but being with my children together 24 hours a day (*…*) they have ADHD and I have been treated for depression";* or problematic situations such as a divorce: *"Everything occurs at once*, *just 15 days ago I started to file for divorce and here we are all living with this additional situation"*. Anyhow, this discourse always focuses on the **loss** experience rather than opportunity, for example: "*I am a very outgoing person and I like to be surrounded by friends"*, *"I am not getting emotionally upset because I try to fill my time as much as possible*, *but I do lack mobility*, *I am not doing well physically”*. Furthermore, the emphasis of the discourse shifts towards the **uncertainty**, rather than the perception of temporariness: *"on the one hand I feel lucky because I am doing well physically and my family is fine*, *on the other hand going shopping and not finding the things that I need makes me feel anxious*, *not being able to go out for a walk to clear my head and*, *worst of all*, *not knowing how long this is going to last"*.

#### Maladjustment: “Not doing well”

Among those who are "not doing well", the adaptation narrative is clearly a path of maladjustment, and is articulated from two core themes: the feeling of uncertainty and the interrupted normality.

Regarding the **uncertainty**, it seems to generate a lot of distress *("feeling overwhelmed"*, *"feeling agony about what may come*, *worried about the virus and the economic future of the country"*), and parents emphasize two uncertain aspects that prevent them from adapting properly to the situation: 1) the job, either because they don’t know what is going to happen or their working activities have stopped ("*I am doing bad*, *I am self-employed in transportation and my major client has paralyzed the orders*. *And I have no business")*, or because working moves them away from taking care of their families *("I am doing bad*, *I work in public health services*, *I have to go to work and leave my daughters alone")*, or because they are finding it hard to adapt to doing home office *(" working from home is hard for me because I don’t have the resources*, *a printer*, *for example");* and 2) the fear of infecting themselves or their family members: *"I leave the house because I have to work in a supermarket*. *But I would rather stay at home because of my fear spreading the virus*, *since my son is population of risk"*, *"I am afraid that someone in my family will be infected"*.

The second basic category in the discourse of maladjustment to confinement is the **interrupted normality,** referring that they are unable to make sense of the situation or establishing new routines: *"I am doing bad*. *Not only because you can’t leave your house*, *but also because of all that it entails (changing your work and personal habits*…*)"*, *"I am doing pretty bad*, *and if you go shopping you are also feeling bad because the city is dead"*. In this discourse, there is no reference to the opportunities of enjoying things that there was no time to do before, as was the case with those who were “doing well".

### Adolescents’ discourse according to perceived outcomes

#### Positive adjustment: “Doing well”

**Fig 2:**
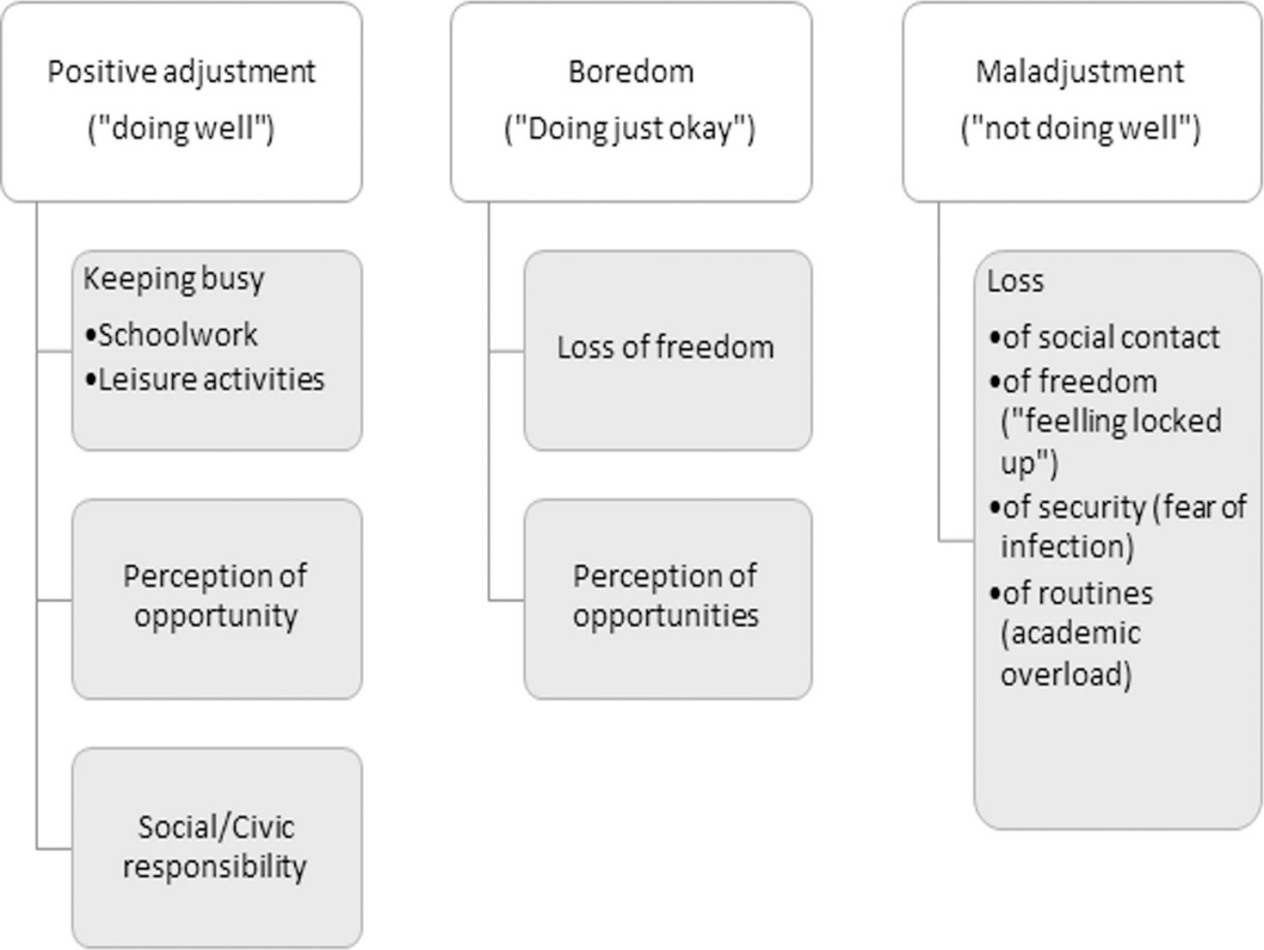
Core themes of adolescents’ discourse according to their perceived adaptation to confinement.

Among those adolescents who say that they are doing "well" or "better than they thought" not leaving the house, the main topic is **keeping busy** in different activities, including studies and leisure activities, either alone or with friends, and always using technology (Fig 2): *"I am doing very well*, *I do my school work in the morning and in the afternoon I use the computer"*, *"It is not a super cool situation but I do many things and I am doing well"*, *"I play online games with my friends"*. This discourse, like the adults, is articulated from the **perception of opportunities** to do or enjoy things that they usually cannot: *"I like it because I don’t have to get up early or go to school and I can play video games every day"*, *"I am doing great*. *I use the time I have to catch up with my studies*, *spend more time with my family and relax more";* and the awareness of their **civic responsibility**, which allows to give meaning to the sacrifice and makes it more bearable: *"Well*, *the truth is that sometimes it is very tiring but then you think we are doing this to save lives and at least It makes me calm down"*, *"I miss my friends and work out*, *but I understand that this is a sanitary emergency that involves all of us*, *and I cannot think selfishly at this time”*. However, the complaints about the schoolwork overload are not uncommon: *"I am overwhelmed by all the homework given by the teachers*, *but in general I am doing fine"*, *"Not going outside is not the problem*, *I am fine with that*, *I have issues with being at home and being sent homework and more homework because it is hard for me to stay calm*, *telling the truth";* and other aspects such as *"I miss going outside and seeing my friends"* or that it is *"a little boring"*.

#### Boredom or moderate adjustment: “Just doing okay”

The discourse of the adolescents who say they are doing "just okay" is similar in certain aspects to the one of those who are doing "well" and, in other aspects, to the one of those who are not doing well or even "bad", but it is mainly articulated from the feeling of boredom and tiredness: *"In the afternoon I get a little bored because in the morning I do my homework*, *if I don’t finish I continue in the afternoon*, *then I watch a movie with my parents"*, *"It’s pretty boring*, *but I do things to keep myself busy";* the overall feeling they express is *"tiredness and exhaustion"*. In addition, the discourse is similar to the one of those who "are not doing well" with regard to the **loss of freedom**: *"I am not doing bad*, *but I am stressed out because the reason I am not leaving the house is not my own choice*, *they are forcing me to*, *and I feel locked in"*. Nevertheless, they also have aspects in common with those who are "doing well", as they find and describe some positive aspects of the situation or **opportunities** to enjoy: *"It is boring not being able to leave the house and I miss my friends and family who do not live with me*, *but we have discovered fun things to do at home*. *Besides*, *I like to go out on the balcony and applaud and see all the neighbors"*.

#### Maladjustment: “Not doing well”

Among the adolescents who are not doing well or even "bad" due to the domestic lockdown, the discourse lacks positive or adaptive aspects regarding the situation, and it articulates from the **perception of different losses** and the suffering they entail, which are related to the above-mentioned complications and complexities (academic overload, fear of infection, social isolation or feeling locked in): *"I’m struggling a bit because I can’t see my grandparents and I have a lot of homework*. *Besides*, *I’m scared because my mother is a pharmacist and is exposed to risk"*, *"I try to handle it well*, *but at the end of the day I collapse and burst in my room by myself"*, *"It’s pretty bad because I used to go out a lot with my friends and now I can’t see them"*, *"I don’t like being locked up with my family*, *I miss my friends and my boyfriend*, *and I need to get outside"*.

### Protection and risk factors

#### Perceived protection factors

Among the perceived protection factors, there can be distinguished two sources: external and internal. External factors refer mainly to the **social support**: *"my family"*, *"my friends"*, *"relate to other people even through video calls"*, *"whatsapp groups"*, *"the company of my children"*, *"the technology that allows me to be in contact with my daughter and other loved ones"*. Besides, there are also some cases referring to the existence of **favorable circumstances** or positive aspects in their lives that do not depend on the person but make things better, easier or more comfortable, such as: *"being healthy"*, *“obviously not having any sick”*, *"being confined to a second home and being able to see the sea”*, *"we can have space when someone needs to be alone*, *and eat on the terrace when the sun rises also helps"*.

The discourse about internal factors is more diverse. For instance, participants refer to **keeping busy** by *"Reading*, *watching TV*, *cooking"*, *"Gymnastics"*, or *"listening to the radio"* according to parents; by *"TV and mobile"*, *"studies and the console"* according to adolescents; stressing that adolescents try more often to keep busy (*Z* = 2. 09; *p* = .03) and that they mention technology more than their mothers and fathers in almost all the activities that help them to keep busy: *"Watching Netflix*, *playing video games*, *making video calls"; "Playing games online with my friends"; "above all technology*: *video games*, *movies*, *etc*. *As a last option I exercise*, *I don’t have weights*, *an exercise bike*… *but I manage with some videos from YouTube and an app"; "the Play (PlayStation Console)"*. Other internal factors that help participants to adapt more easily to the domestic lockdown are **time planning**: *"doing activities*, *being busy*, *keeping routines"* or the *"organization at home"* according to parents; and according to adolescents: *"following a routine"*, *"having a schedule and disconnecting from the TV/information";* perceiving the **opportunities** offered by the situation: *"to rest a lot"*, *"to have time to exercise and share with my children"*, *"we can all enjoy ourselves more*, *which is not possible in everyday life"*, or *"to rethink my life again"* according to the parents; *"to do things that you don’t do in your routine*, *such as playing a little ping-pong with my father*, *playing with my dog*, *etc*. *Things that you normally don’t do"*, *"being able to rest"*, *"redecorating my room"* according to adolescents; activating **personal resources** such as "*thinking that this going to end eventually"*, *"my state of mind"*, *"looking for something positive every day"*, *"patience"*, *"trying to find moments of joy and distraction from the situation*"; according to parents, who refer to this kind of thoughts more than adolescents (*Z* = 2. 28; *p* = .02), who only mention it once: "*I think it is a good way to handle it*: *not to regret being locked up at home without going out*, *but to concentrate on positive and useful things that we like to do"*. In addition, the awareness of **civic responsibility** is another protective factor, which only appears in parents: *"To know that we are protected and doing the right thing*, *and that there is a solution"*, *"To know that it is necessary*, *and it is for the good of my family and mine"*, *"to think that there is no other way to help"*.

#### Perceived risk factors

Among the greatest challenges for the adaptation to the domestic lockdown, participants stress three main topics: the loss experience (of social contact, of mobility, or freedom), negative emotions and problems of cohabitation.

With respect to the **loss of social contact**, parents speak in more general terms, referring to "*Not being able to go and see family/friends"*, but also *"The loss of human contact"*, *"Not seeing people"* as the most difficult issue; while adolescents mention primarily *"Not being able to see friends"*. Regarding the **loss of mobility**, parents also talk more generally and about a wider variety of activities, referring to *"not being able to do sports or walk in the mountains/beach"*, *"walking in the open air*, *not being able to walk in the street"*, *"not getting the sun on my face";* while adolescents primarily complain about sports: *"not being able to swim/walk/play soccer"*. With respect to the **loss of freedom**, parents express significantly greater difficulties than adolescents (*Z* = 3.00, *p* = .003), stressing *"feeling that your freedom has been taken away from you"*, *"I am short of breath*… *in the abstract*, *but it is the awareness of the loss of freedom"*, *"not having a free life";* while only one adolescent mentions *"not being able to walk freely"*.

With regard to to dealing with **negative affects**, the most common negative emotions for parents was fear, accompanied by the feeling of isolation, monotony or *"not being able to find relief*, *to cry*, *to scream*, *to run away …”*. Whereas the most frequent negative emotion for adolescents was boredom: "*I get bored"*, *"you suffocate and feel like going out the window"*, *"you’re stuck behind four walls all day long"*. At last, parents mentioned the **problems of cohabitation** as a challenge to organize living in domestic lockdown: *"The overload that we have at home*, *where we have to do our job*, *support our children*, *especially in distance learning*, *and house chores (meals*, *cleaning etc*.*)"*, *"the management of free time";* and the adolescents, the difficulty to have their own space: *"there are seven of us in a small house*, *not being able to have a little privacy and freedom"*.

## Discussion

There is already abundant evidence that the situation of domestic lockdown and social isolation has been psychologically challenging for adults in different countries, causing depressive or anxious symptoms [4–6, 8, 33–35]. Although there are fewer studies, there is also some evidence on the appearance and increase of these types of symptoms in adolescents [7, 10, 11]. In addition, it had been suggested that families with adolescents are especially vulnerable [13–15]. Therefore, the main objective of this study was to explore the process of adaptation to the lockdown due to COVID-19 of Spanish adolescents and their families that may enhance psychological problems. The specific objectives were analyzing the subjective perception of adolescents and their parents regarding the strategies they used to adjust to the situation of domestic lockdown, the perceived outcomes of the adoption process, and what makes things easier (protection factors) or harder (risk factors) for them.

This study was carried out in the early stages of domestic lockdown in Spain, when the most restrictive measures were established including not leaving home except for a few exceptions. Therefore, these data refer to the first month of adaptation to the national lockdown [2]. In this context, three types of discourse were observed in both adults and adolescents, stating three different types of adaptation. Nevertheless, qualitative differences were observed between both groups. Most of the participants reported a positive adjustment and only 20% of the parents and 16% of the adolescents affirmed that they had not been able to adjust to the lockdown situation and therefore, struggled with psychological distress.

These three different types of adaptation can be identified as: 1) Positive adjustment, 2) Neutral or moderate adjustment, called resignation among parents and boredom among adolescents, and 3) maladjustment. Parents and adolescents who claimed to have had a better adaption to this situation, despite of perceiving some difficulties and suffering some emotional ups and downs, were clearly more focused on positive aspects of the situation. Basically, they applied two strategies: appealing to citizen responsibility (feeling useful in fulfilling the rule of staying at home) and re-discovering ways to enjoy time with the family, being at home or sharing life at a distance (reframing the situation of loss as an opportunity). Nevertheless, the main strategy for parents has been to dedicate time to develop a good family atmosphere, through the organization and planning of routines, which was fostered by already existing comforts such as having a large house, a terrace or a balcony.

Some aspects of the parents’ discourse, such as having a routine and the possibility of looking outside, had previously been identified as protection factors for anxious and depressive symptoms among adults [9]. The need for establishing a routine has probably contributed to parent’s adjustment because it has also been one of their main concerns: how to ensure the well-being of their children and the quality of their studies [17]. Previous research has stressed that families with pre-existing physical or psychological pathologies would have more difficulties to adjust. In the present study, the existence of pre-pandemic problems is only referred to by adults. Some of them adapted poorly to the situation and focused their discourse on the negative aspects of the situation: the loss experiences or the uncertainty inherent to the situation. However, among those parents with pre-existing physical, psychological or social problems more than half of them showed a good adjustment. Thus, although pre-exisiting problems could be considered as a risk factor, the adaptation strategies employed can mitigate their negative effects.

Finally, some parents did not manage to adapt properly to the extraordinary situation. Their maladjustment seems to be related to the inability to manage the uncertainty, especially when associated with work or economic aspects and the fear of infection. It should be noted that in April 2020, when barely a month had passed since the declaration of the global pandemic, the scientific evidence on the mechanisms of infection and how to avoid it was weaker and to some extent, perceived as contradictory. For parents who claim not to have been able to adapt to the domestic lockdown and social isolation, an unmanageable amount of uncertainty was added to that of a disrupted normality. This adverse situation was difficult to assimilate since parents had not been able yet to generate resources for the family organization that promotes a good climate.

In addition to the cognitive reframing processes mentioned above (appealing to the social responsibility and the discovery of opportunities in an adverse situation), the main adaptation strategy for adolescents had been to keep busy with their academic and leisure activities. A previous study had indicated that the adolescents perceived themselves as less anxious and with better coping skills than their parents [16], an observation, which is consistent with the present research. Although there are no significant differences between the number of parents and children who succeeded or failed in the adaptation process, we observed difference on a qualitative level. Thus, those adolescents, who had certain difficulties to adapt completely (“doing just okay”), also manifested qualitatively different psychological distress than did their parents. Like them, adolescents tend to focus on negative aspects such as the loss of freedom or autonomy. This loss experience can be especially harmful in adolescents since the development of an individual identity is one of the main psychosocial tasks in this developmental stage [22]. Instead, the loss of freedom is significantly less mentioned among adolescents compared to their parents. In addition, unlike parents with a moderate adjustment, adolescents were capable of finding positive aspects, evidenced by the perception of opportunities during the lockdown.

Results stress the important role that the social support seems to have for both adolescents and their parents; being perceived as the most frequent protection factor. Previous studies have already suggested its mediating effect on adolescent mental health during lockdown due to COVID-19 [12] and that using the social networks to communicate with loved ones can be effective in mitigating the negative psychological effects of living under severe mobility restrictions [7, 10, 18]. Similarly, spending time with family has been crucial for reducing depressive symptoms in adolescents, probably by providing an important source of social support and contributing to build an adaptive narrative to this extraordinary situation that is perceived as being plagued by losses [22].

Regarding the perception of loss, it seems to be a more dominant experience among adolescents than among parents, especially for those adolescents who report not having been able to adapt to the lockdown situation. The loss experience, which they have reported, referred mainly to: the loss of routines, which leads to academic overload; the loss of social contact and the loss of autonomy or freedom, which makes them feel locked up; but also the loss of security that comes with the fear of infection. These loss experiences imply negative emotions and problems at home that they perceived as the main difficulties for adaptation and may be considered as risk factors. It seems that, at least for adolescents who feel they have failed to adapt to the domestic lockdown, they have experienced a process of grief, which makes no sense. Perhaps the loss of meaning is the fundamental loss, indeed. Our observations suggest the importance of promoting strategies of emotional regulation among adolescence, since emotional intelligence is essential for a positive psychological adjustment [24, 36]. With regard to the loss of security, it has been observed that the lack of routines (such as going to school or work), the duration of the lockdown period, the fear of infection, the lack of information and the lack of personal space at home may represent risk factors for mental health during the domestic lockdown in both adults and adolescents [7, 18]. Those challenges call for new strategies to relate with others, to work and study from home and, finally, to find meaning in daily life. Thus, routines involve the perception of a reliable and safe environment, which is important for adolescent’s relationships and the identity development. Similarly, it has been suggested that social isolation, as well as the adaptation to new behaviors such as wearing a mask, may have a negative impact on their personal and social development [22].

Furthermore, those adolescents, who reported a moderate or maladjustment, explained their negative experience during domestic lockdown mainly by feelings of boredom and tiredness, the overload of schoolwork, the fear of infection, social isolation and feeling locked up. Our results confirm previous scientific literature, which emphasizes boredom and social isolation as the most frequent difficulties experienced by adolescents during lockdown due to COVID-19 [10, 12, 13, 22, 24]. Nonetheless, the present research attempts to offer more in-depth knowledge on the adolescents’ adaptation process during this extraordinary period. Our results suggest that those adolescents, who have been able to discover new opportunities to relate with others and to expand their behavior repertories showed a better adjustment. Our findings provide some insight on how they have dealt with boredom and/or social isolation. Similarly, some previous studies indicate that fewer feelings of boredom and isolation, together with an improved quality of interpersonal communications, reduced the psychological symptoms in confined individuals [18]. Thus, adolescents, who participate in the present research, also mentioned the improvement of positive family relationships as an opportunity, which has been presented by the lockdown situation.

The present research aims to provide a better understanding of the psychological adjustment of adolescents and their parents during the COVID-19 pandemic, an area of research still recent with few published studies [22, 24]. However, our study has some limitations that must be taken into account. First, some characteristics of the sample of this study prevent from generalization of the results, such as all participants being Spanish, with an average socioeconomic level and, for parents, who were mainly women. Nevertheless, a qualitative design was used with exploratory purposes and the understanding of the psychological processes underlying the participants’ discourse is more relevant than the generalization of the results, which does belong to quantitative studies [27, 30, 31]. Secondly, the data collection was adapted to the new reality experienced by the participants, and online participation may have biased the quality of the responses since the conditions of the assessment cannot be fully controlled. In addition, measures were self-reported from a single source; which provides a valuable insight on participant’s subjectivity, improving ecological and pragmatically validity, but obviously diminish data objectivity.

Future research could improve our findings on the adaptation process in situations of restricted mobility and/or social isolation, especially those related to emotional regulation, since they influence the mental health of adolescents and their parents [24]. To begin this future line of research, it may be useful to employ qualitative methodology and open-ended questions, which allow to observe a large and diverse amount of information, not biased by the researchers [30, 31, 37]. This is essential when analyzing the subjective realities and meanings that individuals create in order to adjust to a new and very particular situation such as the global COVID-19 pandemic, due to its regency and the scarcity of research published from this perspective [22]. In addition, longitudinal assessments would be advisable for a greater comprehension regarding the impact of the lockdown measures, the mobility restrictions and the "new normality" on the short- and medium-term development of adolescents. Additionally, it might be relevant to focus on other social groups such as children or elderly, families with a low socioeconomic level, and especially vulnerable groups such as migrants or women, who have been victims of gender violence. The current health situation around the world has affected people in heterogeneous ways and the research should reflect this diversity. Finally, it is interesting to further explore the potential positive effects of domestic lockdown and social isolation, such as the re-discovery of oneself and one’s family [22]; enjoying the additional time that is usually scarce; as well as its influence on personal and family resilience to cope with this already extraordinary and unpredictable situation.

## Conclusions

The outbreak and rapid spread of COVID-19 has changed the way of life of millions of people around the world. Although the unprecedented impact on mental health is still being studied in detail, there is some evidence in this regard [3–8, 10, 11, 18, 33–35], and more and more studies stress the importance of exploring not only the quantitative effects but also the individual adaptation process and subjective perceptions, especially among families with adolescent children [15, 17, 22, 24].

Understanding the adaptation process during the early stages of domestic lockdown is a key step in designing effective assessment, prevention and intervention tools to enhance the mental health and psychological well-being of families, especially for those who reported not being able to develop a positive adjustment. The main contribution of this research consists in observing three cognitive and affective discourses according to the adaptation process perceived by the participants. Our findings provide a deeper understanding of the key factors of positive adjustment in the context of the first month of national lockdown experienced by Spanish adolescents and their parents: some internal or psychological resources such as the awareness of civic responsibility, emotional intelligence, perception of the temporariness of the situation, and the search for opportunities in adverse situations; and some external, such as social support. The most useful strategy for parents was the organization of routines and family spaces; whereas for adolescents was to keep busy. Finally, results suggest that parents who failed to adapt to the extraordinary situation were mainly struggling with the uncertainty. Adolescents, who referred maladjustment, experienced the lockdown mainly as a mourning process, stressing that may be useful to focus on the loss experience and to carefully and longitudinally evaluate the impact of social on their development.

## Supporting information

S1 Dataset Working dataset for mixed-method study. (XLSX)
